# Detection and Characterization of *RB1* Mosaicism in Patients With Retinoblastoma Receiving cfDNA Test

**DOI:** 10.1001/jamaophthalmol.2025.1079

**Published:** 2025-05-08

**Authors:** Chuan Gao, Juber Patel, Melissa Robbins, Erika Gedvilaite, Anita S. Bowman, Kanika Arora, Chad Vanderbilt, A. Rose Brannon, Danielle N. Friedman, Jennifer Kennedy, Elise Fiala, Ozge Ceyhan-Birsoy, Ira J. Dunkel, Britta Weigelt, Jasmine H. Francis, Jorge S. Reis-Filho, David H. Abramson, Diana Mandelker

**Affiliations:** 1 Department of Pathology and Laboratory Medicine, Memorial Sloan Kettering Cancer Center, New York, New York; 2 Department of Surgery, Memorial Sloan Kettering Cancer Center, New York, New York; 3 Department of Pediatrics, Memorial Sloan Kettering Cancer Center, New York, New York; 4 Department of Medicine, Memorial Sloan Kettering Cancer Center, New York, New York; 5 Department of Ophthalmology, Weill Cornell Medical Center, New York, New York; 6 Cancer Biomarker Development, Oncology Research and Development, AstraZeneca, Gaithersburg, Maryland

## Abstract

**Importance:**

Plasma cell-free DNA (cfDNA) testing is increasingly used for disease diagnosis and monitoring in retinoblastoma, with *RB1* allele fraction in cfDNA actively corresponding to disease status and treatment response. However, while *RB1* mosaicism has been reported in retinoblastoma, its clinical implications and potential impact on cfDNA testing remain unclear.

**Objectives:**

To identify *RB1* mosaicism using paired plasma and buffy coat (containing lymphocytes, monocytes, granulocytes, and platelets) DNA testing, and to characterize the implications of *RB1* mosaicism on cfDNA testing outcomes.

**Design, Setting, and Participants:**

In this cross-sectional study, participants with retinoblastoma underwent testing with MSK-ACCESS (Memorial Sloan Kettering–Analysis of Circulating cfDNA to Examine Somatic Status), a clinical assay that combines plasma cfDNA and buffy coat genomic DNA sequencing, enabling the detection and differentiation of somatic, heterozygous, and mosaic variants, between July 2020 and April 2024 at the Memorial Sloan Kettering Cancer Center. Mosaic findings from MSK-ACCESS were correlated with those from a subgroup of patients who concurrently underwent testing using the MSK-IMPACT germline assay. Data analysis was performed from April to September 2024.

**Exposure:**

*RB1* mosaicism in retinoblastoma.

**Main Outcomes and Measures:**

The *RB1* variant allele fractions in cfDNA and buffy coat genomic DNA were used to detect *RB1* mosaicism.

**Results:**

A total of 136 consecutive patients with retinoblastoma (median age at diagnosis, 1.0 year [IQR, 0.4-1.7 years]; 74 [54.4%] female; 67 with bilateral disease and 69 with unilateral disease) who underwent testing with the MSK-ACCESS assay were included. *RB1* mosaicism was identified in buffy coat DNA from 20 patients (14.7%), with consistent results detected in all 11 participants tested concurrently by the MSK-IMPACT (Memorial Sloan Kettering–Integrated Mutation Profiling of Actionable Cancer Targets) germline assay. Four participants with *RB1* mosaicism previously tested negative for germline *RB1* variants by external laboratories. Compared with heterozygous participants, participants with *RB1* mosaicism had a lower risk of developing bilateral disease (91.7% vs 55.0%, respectively; difference, 36.7% [95% CI, 13.8%-59.6%]; *P* = .002). In cfDNA, the mosaicism variant was detected both before and after treatment, with variant allele fraction initially decreasing after treatment but then stabilizing at levels consistent with mosaicism, despite the absence of clinical disease.

**Conclusions and Relevance:**

The accurate detection and quantification of *RB1* mosaicism are crucial. *RB1* mosaicism should be considered when *RB1* variants persist in cfDNA after treatment without evidence of disease; failure to do so may lead to false-positive results and overtreatment in patients with *RB1* mosaicism. Identifying *RB1* mosaicism may improve patient counseling, inform treatment decisions, and enhance surveillance efforts.

## Key Points

**Question:**

Can a cell-free DNA (cfDNA) assay, when paired with buffy coat genomic DNA testing, detect *RB1* mosaicism, and what are the implications for cfDNA testing outcomes?

**Findings:**

In 136 participants with retinoblastoma tested by the MSK-ACCESS (Memorial Sloan Kettering–Analysis of Circulating cfDNA to Examine Somatic Status) assay, 20 (14.7%) had *RB1* mosaicism at variant allele fractions as low at 1%; unlike somatic variants, mosaic *RB1* variants persisted in cfDNA after treatment even in the absence of clinical disease. Participants with *RB1* mosaicism had a lower risk of bilateral disease compared with heterozygous participants.

**Meaning:**

These findings support consideration of *RB1* mosaicism to reduce false-positive cfDNA result interpretations, potentially enhancing risk assessment.

## Teaser

This cross-sectional study identifies and characterizes *RB1* mosaicism in patients with retinoblastoma using a clinical assay that combines plasma cell-free DNA (cfDNA) testing and buffy coat genomic DNA testing.

## Introduction

Cell-free DNA (cfDNA) is defined as extracellular DNA that can be released from both healthy and diseased cells. In patients with cancer, tumor DNA is released into the circulation through apoptotic and necrotic processes characteristic of tumor cells, making cfDNA valuable material for tumor-derived variant profiling.^1,2^ The buffy coat is a thin layer of cells that forms between the plasma and red blood cells after centrifugation of whole blood. This layer contains a concentrated mixture of white blood cells (including lymphocytes, monocytes, granulocytes, and platelets) and reliably provides a high-yield source of genomic DNA for genetic analysis.^3^

MSK-ACCESS (Memorial Sloan Kettering–Analysis of Circulating cfDNA to Examine Somatic Status) is a next-generation sequencing (NGS) assay that profiles tumor variants based on cfDNA from peripheral blood samples.^4^ In addition, the assay incorporates buffy coat–derived DNA sequencing, allowing the detection and differentiation of both somatic and germline variants while ruling out clonal hematopoiesis. With high-depth coverage, it may open opportunities to detect mosaic variants with low variant allele fractions (VAFs).

Retinoblastoma is the most common primary childhood eye cancer, affecting approximately 6 in 100 000 live births.^5^ Approximately 98% of retinoblastomas are initiated by *RB1* loss-of-function variants, which can be somatic, germline heterozygous, or mosaic.^5,6^ Studies have indicated that tumor-derived *RB1* variants can be effectively detected in plasma cfDNA of patients with active retinoblastoma.^7,8^ In recent studies, researchers observed a decremental decrease of *RB1* cfDNA VAF in patients with somatic retinoblastoma following intra-arterial chemotherapy, suggesting that relative VAF changes could serve as a biomarker of treatment response and disease status.^9,10^ However, while mosaicism is a well-known mechanism in retinoblastoma,^6,11^ the potential implications of *RB1* mosaicism in plasma cfDNA testing are, to our knowledge, not well studied.

In this study, we aimed to detect *RB1* mosaicism in a cohort of participants with retinoblastoma using a buffy coat DNA–paired cfDNA assay and to characterize its risk of bilateral disease. More importantly, the potential implications of *RB1* mosaicism on cfDNA-based testing were investigated.

## Methods

All study participants were enrolled in a research protocol (ClinicalTrials.gov identifier: NCT01775072) approved by the Memorial Sloan Kettering Cancer Center institutional review board. Written informed consent for somatic and germline genetic analysis was obtained. Participants did not receive compensation for this study. This study follows the Strengthening the Reporting of Observational Studies in Epidemiology (STROBE) reporting guidelines. Data analysis was performed from April to September 2024.

The participants underwent MSK-ACCESS testing between July 2020 and April 2024, involving the analysis of both cfDNA and buffy coat–derived genomic DNA. When available, participants were tested multiple times, including both before and after treatment. A detailed description of MSK-ACCESS has been previously published.^4^ In short, MSK-ACCESS is a paired assay combining plasma cfDNA and genomic DNA derived from white blood cells in the buffy coat. Hybridization capture was performed, followed by paired-end sequencing, allowing the detection of very low-fraction alterations. This assay identifies multiple classes of genomic abnormalities, including single-nucleotide variants (SNVs), insertions or deletions (indels), and copy number alterations. Probes were designed to cover 129 genes, including the entire length of all exons in the *RB1* gene. The matching of cfDNA and white blood cell DNA was confirmed using a set of fingerprint single-nucleotide polymorphisms (SNPs). Mosaic *RB1* variants were correlated and confirmed separately by MSK-IMPACT (Memorial Sloan Kettering–Integrated Mutation Profiling of Actionable Cancer Targets), a clinically validated tumor-normal matched assay that detects mosaicism from whole-blood DNA.^12^ Both MSK-ACCESS and MSK-IMPACT were approved for clinical use by the New York State Department of Health. Germline variants detected from the buffy coat DNA were classified according to American College of Medical Genetics and Genomics guidelines.^13^

Genetic ancestry was inferred for each participant as previously described.^14^ Briefly, more than 3000 autosomal SNPs from the 1000 Genomes Project were included for a supervised model by ADMIXTURE.^15,16^ Genetic ancestry was computed to the proportions of African, East Asian, European, Native American, and South Asian populations. Participants were assigned to a single population ancestry if their ancestry proportion was greater than 0.8 for that population. Participants with an ancestry proportion less than 0.8 for any single population were classified as admixed. Ashkenazi Jewish ancestry was inferred using 282 additional SNP markers as previously described.^14^

### Statistical Analysis

Statistical analysis between mosaic and heterozygous participants was performed using a 2-sample *t* test. Percentage confidence intervals were computed using a binomial test. All analyses were computed using R version 4.3.2 statistical software (R Foundation).

## Results

### Participant Characteristics

**Figure 1.**
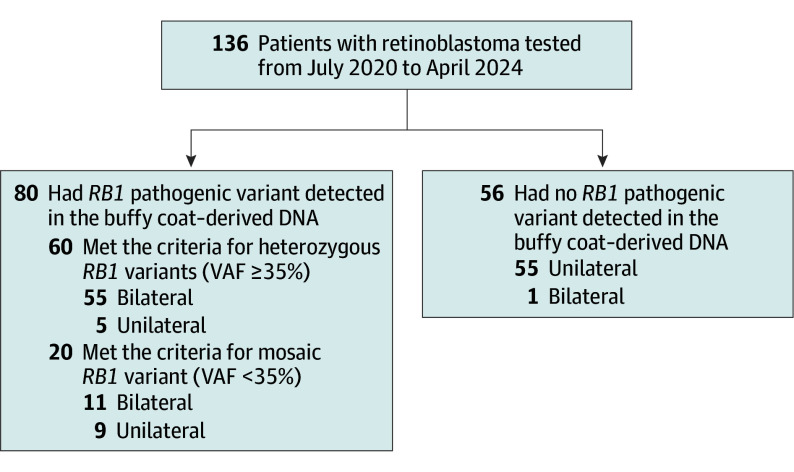
Participant Counts of the Study Cohort Participants with sequence variants with a variant allele fraction (VAF) between 25% and 35% were suspected to have a mosaic variant. Participants without an *RB1* pathogenic variant in buffy coat–derived DNA were considered to have disease of somatic origin, although a germline variant outside the assayed region cannot be ruled out.

This study included 136 consecutive patients with retinoblastoma (74 [54.4%] female) tested with MSK-ACCESS between July 2020 and April 2024 (Figure 1). The median age at diagnosis was 1.0 year (IQR, 0.4-1.7 years), with the youngest participant diagnosed when younger than 1 month. The cohort was ethnically diverse, with more than half of the participants (72 [52.9%]) coming from non-European genetic ancestries, including admixed, African, Asian, and Native American populations (eTable 1 in Supplement 1).

### *RB1* Variant Detection in Buffy Coat DNA

Pathogenic *RB1* variants were detected in buffy coat–derived DNA in 80 (58.8%) of the 136 participants, with VAFs ranging from 1.2% to 64.3%, indicating the presence of a germline heterozygous or mosaic variant. The remaining 56 participants (41.2%), without a detected *RB1* variant in the buffy coat DNA, likely had somatic disease, although the possibility of deep intronic *RB1* variants outside the tested regions cannot be ruled out.

To further characterize the VAF distribution of germline heterozygous variants and define the VAF cutoff for mosaic variants, 163 heterozygous variants (49 SNVs and 114 indels) from other genes on the panel were analyzed. These variants were confirmed as heterozygous through MSK-IMPACT. The minimum VAF for the tested heterozygous variants was greater than 36.0%, with average VAFs exceeding 48.0% for both SNVs and indels and with standard deviations ranging from 0.02 to 0.04 depending on the variant type and size (eFigure 1 in Supplement 1). On average, heterozygous indel VAFs were more variable than SNV VAFs. Therefore, *RB1* SNVs with VAFs below 35.0% and indels below 25.0% in the buffy coat DNA were classified as mosaic; indels with VAFs between 25.0% and 35.0% were considered suspected mosaic. Variants with higher VAFs were classified as heterozygous (eFigure 2 in Supplement 1).

Within the cohort, 60 participants met the criteria for heterozygous variants (VAF ≥35.0%), while 20 participants had *RB1* variants classified as mosaic (VAF <35.0%, ranging from 1.2%-34.7%), including 5 suspected mosaic indels (Table). Additionally, 12 of the 20 participants with *RB1* mosaicism underwent multiple testing (a mean of 3.50 tests [95% CI, 2.33-4.67] per participant), with mosaic variants consistently detected at similar VAFs (eTable 2 in Supplement 1).

**Table.** Summary of Participants With the Mosaic Variant or Suspected Mosaic Variant

Participant No.	Age at diagnosis, y/sex	Laterality	Sequence change		Specimens analyzed, No.	VAF, mean (range), %	
			Coding DNA	Protein		Buffy coat DNA	cfDNA
1	3.2/F	OU	c.1060_1061del	p.Q354Efs*7	3	4.39 (3.77-5.41)	2.59 (2.05-3.12)
2	1.3/M	OU	c.1072C>T	p.R358*	2	15.53 (14.27-16.79)	11.86 (9.67-14.04)
3	8/M	OS	c.191T>G	p.L64*	1	11.85 (11.85-11.85)	12.51 (12.51-12.51)
4	0.3/F	OU	c.1735C>T	p.R579*	1	15.61 (15.61-15.61)	12.43 (12.43-12.43)
5	0.6/F	OU	c.2359C>T	p.R787*	2	29.79 (28.77-30.80)	25.57 (25.14-25.99)
6^a^	1.3/F	OU	c.2237_2241del	p.E746Gfs*3	1	34.66 (34.66-34.66)^a^	33.26 (33.26-33.26)
7^a^	1.4/F	OU	c.2125_2135dup	p.K713Mfs*6	3	30.84 (30.2-31.69)^a^	31.45 (30.32-32.28)
8	1.6/F	OS	c.964G>T	p.E322*	1	14.71 (14.71-14.71)	15.33 (15.33-15.33)
9	2.4/F	OU	c.958C>T	p.R320*	2	14.94 (12.98-16.89)	14.38 (11.12-17.64)
10^a^	0.6/F	OS	c.2429_2432dup	p.S811Rfs*5	1	28.50 (28.50-28.50)^a^	24.02 (24.02-24.02)
11	4/F	OU	c.2363_2384dup	p.R798Qfs*4	1	8.03 (8.03-8.03)	8.19 (8.19-8.19)
12	1.7/F	OS	c.1183C>T	p.Q395*	8	3.58 (2.67-5.14)	3.15 (2.38-4.37)
13	4.7/M	OS	c.1330C>T	p.Q444*	2	4.41 (4.24-4.58)	4.19 (4.15-4.22)
14	2/M	OU	c.1174del	p.A392Qfs*9	5	17.97 (17.21-18.33)	18.64 (17.54-19.63)
15^a^	1.7/M	OU	c.45_76del	p.A17Pfs*3	3	31.13 (29.23-32.39)^a^	39.80 (36.64-41.74)
16^a^	0.7/M	OU	c.2073_2075dup	p.Y692*	4	33.43 (32.11-34.68)^a^	29.55 (27.63-31.52)
17	1/F	OD	c.1399C>T	p.R467*	8	10.11 (8.21-12.03)	11.14 (8.00-26.25)
18	0.4/M	OS	c.1735C>T	p.R579*	3	4.92 (4.52-5.19)	4.30 (4.26-4.37)
19	1/M	OD	c.2359C>T	p.R787*	1	1.21 (1.21-1.21)	2.96 (2.96-2.96)
20	0.9/F	OD	c.54_76dup	p.P26Rfs*47	1	12.08 (12.08-12.08)	17.02 (17.02-17.02)

Abbreviations: cfDNA, cell-free DNA; F, female; M, male; OD, right eye; OS, left eye; OU, each eye; VAF, variant allele fraction.

^a^ Suspected mosaic *RB1* variant (insertion or deletion with VAF of 25%-35%).

Among the 20 participants with *RB1* mosaicism, 11 also underwent germline testing by MSK-IMPACT. All of the 11 participants had the same mosaic finding by MSK-IMPACT. Analysis of the VAFs also revealed comparable VAFs between MSK-ACCESS and MSK-IMPACT, except for 1 participant (participant 11, VAF 30.8% and 8.0% in MSK-IMPACT and MSK-ACCESS, respectively) (eTable 3 in Supplement 1).

Moreover, 15 participants with *RB1* mosaicism had also been previously tested for germline *RB1* variants by external clinical laboratories between 2009 and 2020. Of these, 11 had the same *RB1* mosaic variant identified, although only 4 were correctly reported as mosaic (participants 1, 2, 4, and 17; eTable 3 in Supplement 1)—the other 7 cases either were misclassified as heterozygous or had no zygosity information provided. Notably, the remaining 4 (26.7%) of the 15 participants with *RB1* mosaicism were reported by external laboratories as being negative for *RB1* variants, despite positive findings by MSK-ACCESS (participants 3, 12, 13, and 14; VAFs 3.6%-18.0%; eTable 3 in Supplement 1).

### *RB1* Variant Detection in Plasma cfDNA

Among the 136 participants, 110 had oncogenic *RB1* variants detected in cfDNA via MSK-ACCESS. As expected, all heterozygous and mosaic variants identified in buffy coat DNA were also present in cfDNA (Table). Negative cfDNA results in the remaining 26 participants were likely due to sample collection after remission in somatic disease. In rare cases, it could also be due to retinoblastoma with *MYCN* amplification with wild-type *RB1* (*MYCN*^A^*RB1*^+/+^).

**Figure 2.**
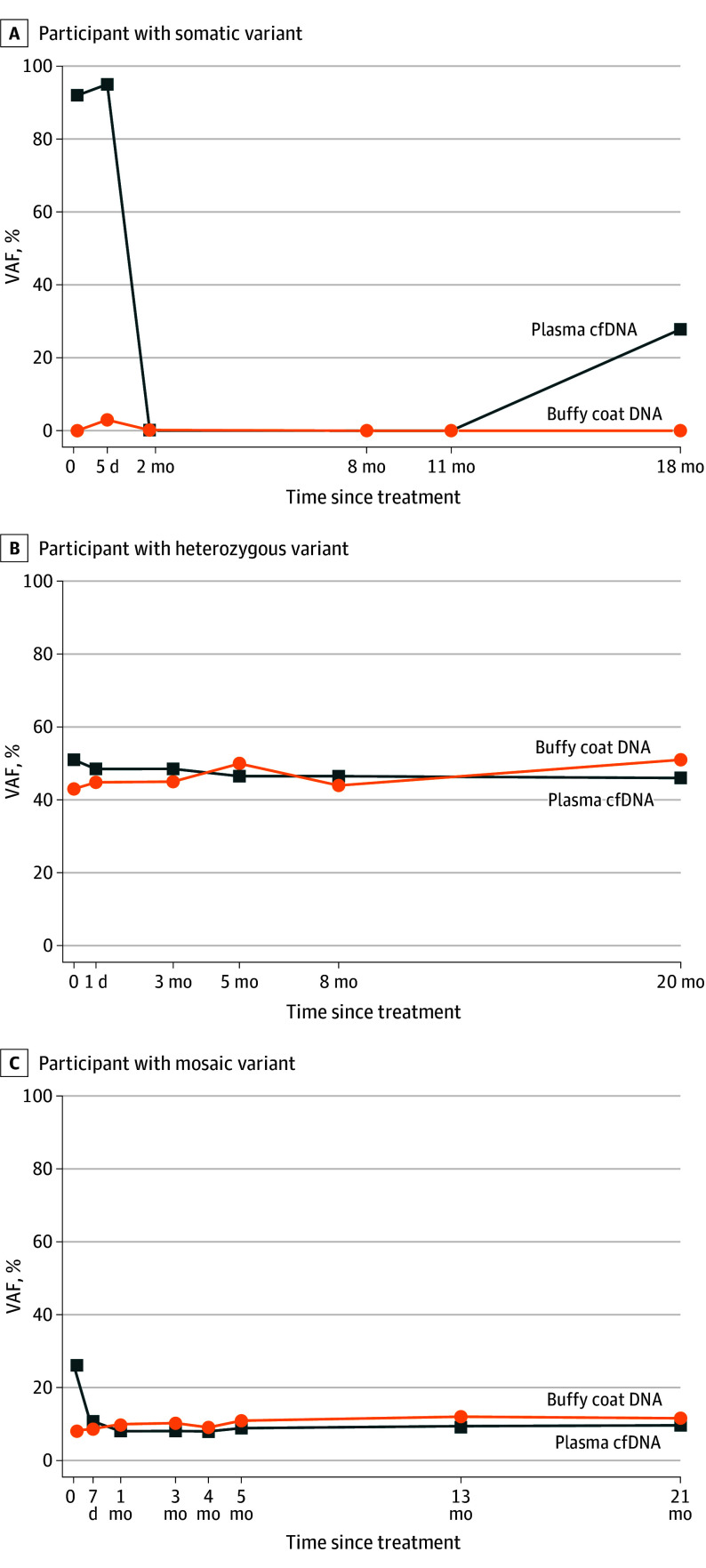
Variant Allele Fractions (VAFs) in Patients With Retinoblastoma VAFs in plasma cell-free DNA (cfDNA) and buffy coat DNA are shown for patients with retinoblastoma due to somatic (A), heterozygous (B), and mosaic (participant 17) (C) variants.

*RB1* VAFs in cfDNA decrease after disease treatment. In the represented participant with somatic disease (Figure 2A), cfDNA VAF started very high prior to the treatment (92.0%) but rapidly decreased below detection limit (0%) after treatment and remained undetectable until a disease relapse (difference, 92.0%; *P* < .001). The cfDNA VAFs from the representative germline heterozygous participant stabilized around 47.8%, which is close to the expected fraction of a germline heterozygous variant at 50% (Figure 2B). In the representative participant with *RB1* mosaicism, cfDNA VAFs initially declined after treatment but then stabilized at levels close to the *RB1* mosaic variant, even in the absence of active disease (end-point cfDNA VAF, 9.7%; mosaicism level, 10.1%; difference, 0.4% [95% CI, −0.6% to 1.4%]; *P* = .43) (Figure 2C; eTable 2 in Supplement 1).

### *RB1* Mosaicism and Disease Laterality

**Figure 3.**
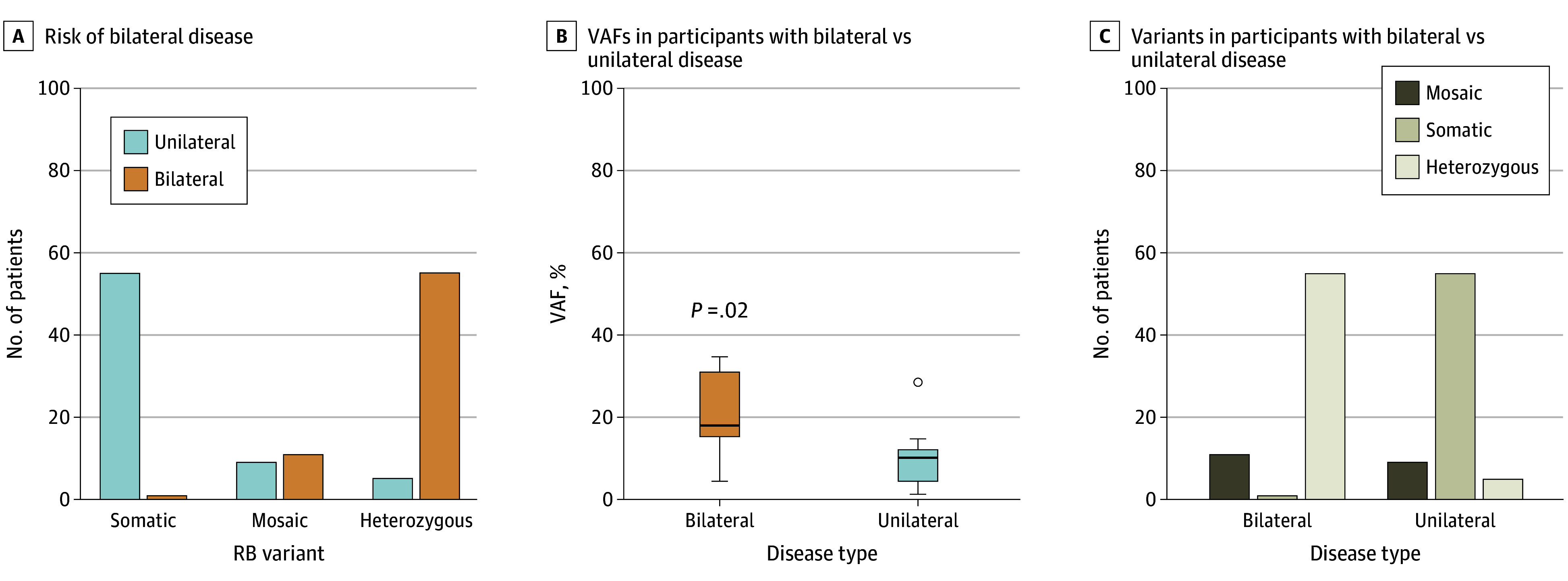
Risk, Variant Allele Fraction (VAF), and Variant Types by Unilateral vs Bilateral Retinoblastoma (RB) A, Participants with a heterozygous *RB1* variant had the highest risk of developing bilateral disease (55 of 60 participants [91.7%]). In contrast, only 1 of 56 participants (1.8%) with a somatic variant developed bilateral disease. Participants with the mosaic variant showed a lower risk for bilateral disease (11 of 20 participants [55.0%]). B, Among participants with the mosaic variant, those with bilateral disease had a higher VAF than those with unilateral disease. The horizontal line within the boxes indicates the median; lower and upper borders of boxes, first and third quartiles, respectively; whiskers, smallest (lower whisker) and largest (upper whisker) values within 1.5 × IQR; circle, outlier. C, The mosaic variant was found in 9 of 69 participants (13.0%) with unilateral disease and 11 of 67 participants (16.4%) with bilateral disease.

In this cohort, 67 of 136 participants (49.3%) had bilateral disease. As expected, participants with a heterozygous *RB1* variant had the highest risk of developing bilateral disease (55 of 60 participants [91.7%]). In contrast, only 1 of 56 participants (1.8%) without detectable *RB1* variants in the buffy coat developed bilateral disease. Participants with *RB1* mosaicism showed a lower risk for bilateral disease compared with heterozygous patients (55.0% vs 91.7%; difference, 36.7% [95% CI, 13.8%-59.6%]; *P* = .002) (Figure 3A and Table). Participants with *RB1* mosaicism who had bilateral disease had higher mean VAFs compared with those with unilateral disease (mean [SD], 21.5% [10.3%] vs 10.2% [7.8%]; difference, 11.3% [95% CI, 2.4%-20.3%]; *P* = .02) (Figure 3B), suggesting a potential VAF-dependent association.

On average, participants with heterozygous *RB1* variants were diagnosed with retinoblastoma at a younger age compared with participants with somatic disease (mean [SD], 0.7 [0.6] vs 1.8 [1.7] years; difference, 1.1 [95% CI, 0.6-1.6] years; *P* < .001) and those with *RB1* mosaicism (mean [SD], 0.7 [0.6] vs 1.9 [1.8] years; difference, 1.2 [95% CI, 0.6-1.7] years; *P* < .001) (eTable 1 in Supplement 1). Of the 67 bilaterally affected participants, 55 (82.1% [95% CI, 70.8%-90.4%]) carried a heterozygous *RB1* variant, and 11 (16.4% [95% CI, 8.5%-27.5%]) had *RB1* mosaicism (Figure 3C). Among the 69 participants with unilateral disease, 9 (13.0% [95% CI, 6.1%-23.3%]) carried a mosaic *RB1* variant, and only 5 (7.2% [95% CI, 2.4%-16.1%]) were heterozygous (difference, 5.8% [95% CI, −4.2% to 15.8%]; *P* = .26) (Figure 3C). No difference on *RB1* mosaicism prevalence was observed between unilateral (13.0% [95% CI, 6.1%-23.3%]) and bilateral (16.4% [95% CI, 8.5%-27.5%]) groups (difference, 3.4% [95% CI, −15.3% to 8.5%]; *P* = .58).

## Discussion

Genetic mosaicism is a well-recognized feature of several monogenic genetic disorders, including retinoblastoma.^6,11^ However, most genetic testing assays are designed to detect germline heterozygous variants with VAFs around 50%, making the detection and accurate quantification of mosaic variants challenging. In this study, we evaluated MSK-ACCESS, an NGS-based clinical assay that pairs plasma cfDNA with buffy coat genomic DNA deep sequencing, in a cohort of 136 participants with retinoblastoma. Our analysis identified 20 participants with mosaic *RB1* variants.

Compared with traditional approaches, MSK-ACCESS demonstrated several advantages in detecting mosaicism. First, the assay uses high-depth NGS of buffy coat genomic DNA with paired-end reads, enabling the detection of mosaic SNVs and small indels with VAFs as low as 1%. Traditional testing methods frequently misclassify mosaic variants as heterozygous or miss them altogether.^17^ For instance, among the 15 participants with *RB1* mosaicism who were previously tested by clinical laboratories, 4 (26.7%) were reported as being negative. Participant 3, who was tested in 2009 using Sanger sequencing and quantitative polymerase chain reaction, missed the mosaic variant (VAF 11.8%), which is unsurprising given the limitations of those methods. Also concerning, however, were the 3 participants (participants 12-14) tested between 2019 and 2020 using NGS and multiplex ligation-dependent probe amplification. Despite the use of more modern technologies, these assays missed mosaic variants at VAFs of 3.6%, 4.4%, and 18.0%, respectively. This underscores the importance of high-depth coverage and paired sequencing of both buffy coat DNA and plasma cfDNA in detecting mosaicism.

One of the major benefits of MSK-ACCESS is its minimally invasive nature, which facilitates repeated testing. This could enable real-time monitoring of disease status, treatment response, and relapse. Previous studies have observed that cfDNA *RB1* VAF decreases following intra-arterial chemotherapy, suggesting that VAF changes in cfDNA can serve as biomarkers for treatment response.^9^ Our results confirmed the potential utility of cfDNA-based assays for monitoring patients with retinoblastoma. However, caution is needed when interpreting results in patients with *RB1* mosaicism, as the presence of mosaic variants in cfDNA can complicate interpretations. For example, in participant P17, cfDNA VAF initially decreased following treatment but stabilized at approximately 10%, consistent with the mosaic VAF in buffy coat DNA (Figure 2C). Without the detection and accurate quantification of mosaicism, this could be misinterpreted as residual disease. Adjusting the end-point VAF for patients with *RB1* mosaicism could be an important component to avoid false-positive findings and overtreatment.

Accurate detection and quantification of *RB1* mosaicism may also have important implications for prognosis and patient management. In our cohort, participants with *RB1* mosaicism had a lower risk of developing bilateral disease compared with germline heterozygous participants. Furthermore, the risk of bilateral disease in participants with *RB1* mosaicism appeared to correlate with the level of mosaicism, with higher mosaicism associated with increased risk. These findings could allow clinicians to offer more personalized counseling and care to patients with mosaic retinoblastoma based on *RB1* variant VAF. Additionally, mosaic variants were present in both participants with unilateral disease and those with bilateral disease at similar frequencies (13.0% and 16.4%, respectively; *P* = .58), supporting the need for *RB1* mosaicism screening in both groups.

### Limitations

There are limitations to this study. While we evaluated VAFs of confirmed heterozygous variants, differentiating between heterozygous and mosaic variants remains challenging, particularly for indels with borderline VAFs. NGS technologies can preferentially amplify or capture the normal homologue, which may skew VAFs for certain indels.^18^ As a result, some high-level mosaic indels classified as suspected mosaic may in fact be heterozygous. Moreover, the genetic mechanisms underlying mosaicism are complex and often involve tissue specificity.^19^ Specifically, the retina is derived from the ectoderm, while blood is mesoderm derived; mosaicism that occurs after germ layer differentiation would not be detectable by peripheral blood–based assays. Furthermore, this study does not determine definitively if having this information vs not having this information changes the morbidity or mortality of patients with retinoblastoma.

## Conclusions

This study evaluated 136 participants with retinoblastoma and demonstrated that MSK-ACCESS, a cfDNA and buffy coat DNA-matched clinical assay, can detect mosaic *RB1* variants with VAFs as low as 1%. Furthermore, our results suggest that cfDNA VAFs hold potential as biomarkers for monitoring disease status and treatment response. The accurate detection and quantification of *RB1* mosaicism offer opportunities for improved counseling, treatment planning, and surveillance. Finally, these results support consideration of evaluating patients with retinoblastoma undergoing cfDNA testing with persistent *RB1* variants in cfDNA but no clinical evidence of disease for *RB1* mosaicism to reduce the chance of a false-positive interpretation or overtreatment.

## Notes

Supplement 1. **eFigure 1.** Variant Allele Fraction Grouped by Type and Insertion/Deletion (INDEL) Size **eFigure 2.** Distribution of Variant Allele Fraction (VAF) of *RB1* Variants **eTable 1.** Demographic Table of Study Participants **eTable 2.** List of All Specimens With Mosaic *RB1* Variants **eTable 3.** Summary of MSK-IMPACT and External Testing Results for the Identified Mosaic Patients

Supplement 2. Data Sharing Statement
