# Supplementary material for: Detection and Characterization of RB1 Mosaicism in Patients With Retinoblastoma Receiving cfDNA Test
Source: JAMA Ophthalmol. 2025 May 8;143(7):562–8. doi: 10.1001/jamaophthalmol.2025.1079 (PMC12062978; doi:10.1001/jamaophthalmol.2025.1079)
Supplement: Supplement 1. — eFigure 1. Variant Allele Fraction Grouped by Type and Insertion/Deletion (INDEL) Size eFigure 2. Distribution of Variant Allele Fraction (VAF) of RB1 Variants eTable 1. Demographic Table of Study Participants eTable 2. List of All Specimens With Mosaic RB1 Variants eTable 3. Summary of MSK-IMPACT and External Testing Results for the Identified Mosaic Patients [file jamaophthalmol-e251079-s001.pdf]

Supplemental Online Content

Gao C, Patel J, Robbins M, et al. Detection and characterization of *RB1* mosaicism in patients with retinoblastoma receiving cfDNA test. *JAMA Ophthalmol*. Published online May 8, 2025. doi:10.1001/jamaophthalmol.2025.1079

eFigure 1. Variant Allele Fraction Grouped by Type and Insertion/Deletion (INDEL) Size
eFigure 2. Distribution of Variant Allele Fraction (VAF) of *RB1* Variants
eTable 1. Demographic Table of Study Participants
eTable 2. List of All Specimens With Mosaic *RB1* Variants
eTable 3. Summary of MSK-IMPACT and External Testing Results for the Identified Mosaic Patients

This supplemental material has been provided by the authors to give readers additional information about their work.

© 2025 Gao C et al. *JAMA Ophthalmology*.

eFigure 1. Variant Allele Fraction Grouped by Type and Insertion/Deletion (INDEL) Size

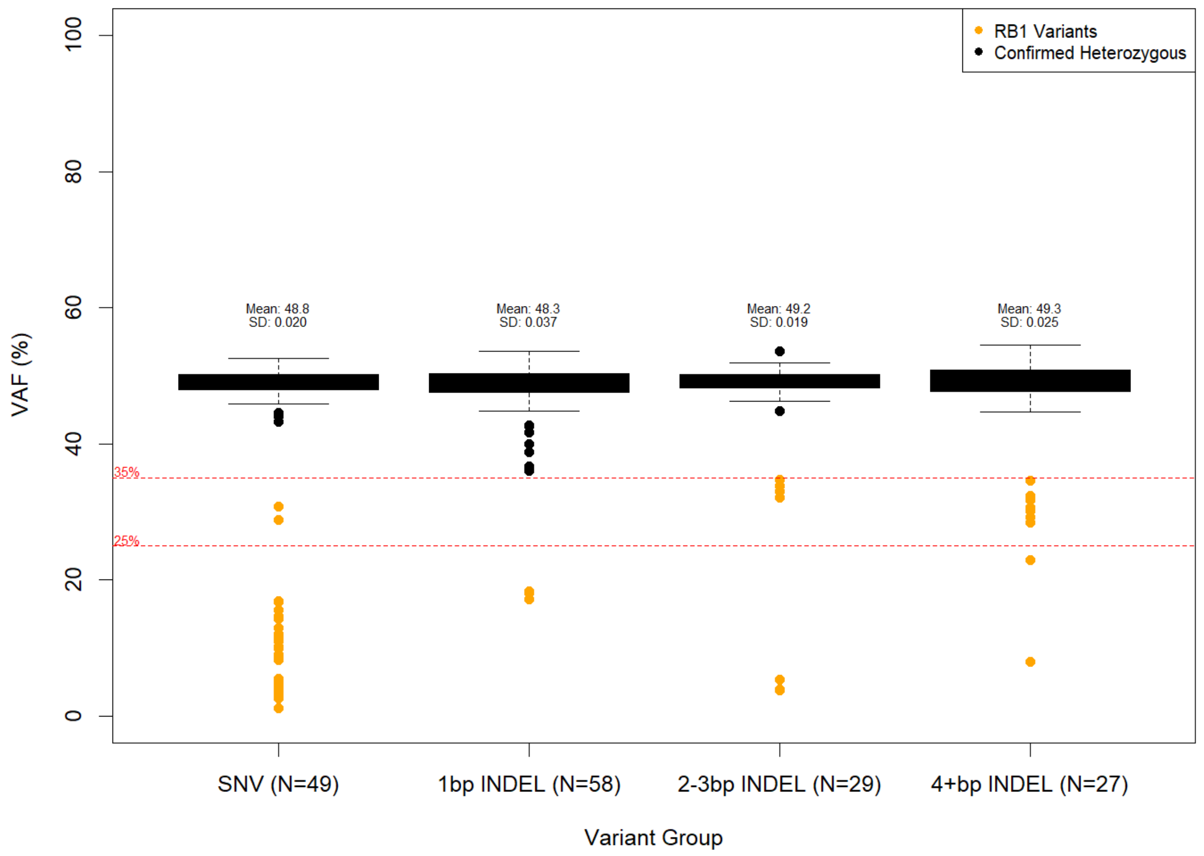

© 2025 Gao C et al. *JAMA Ophthalmology*.

eFigure 2. Distribution of Variant Allele Fraction (VAF) of *RB1* Variants

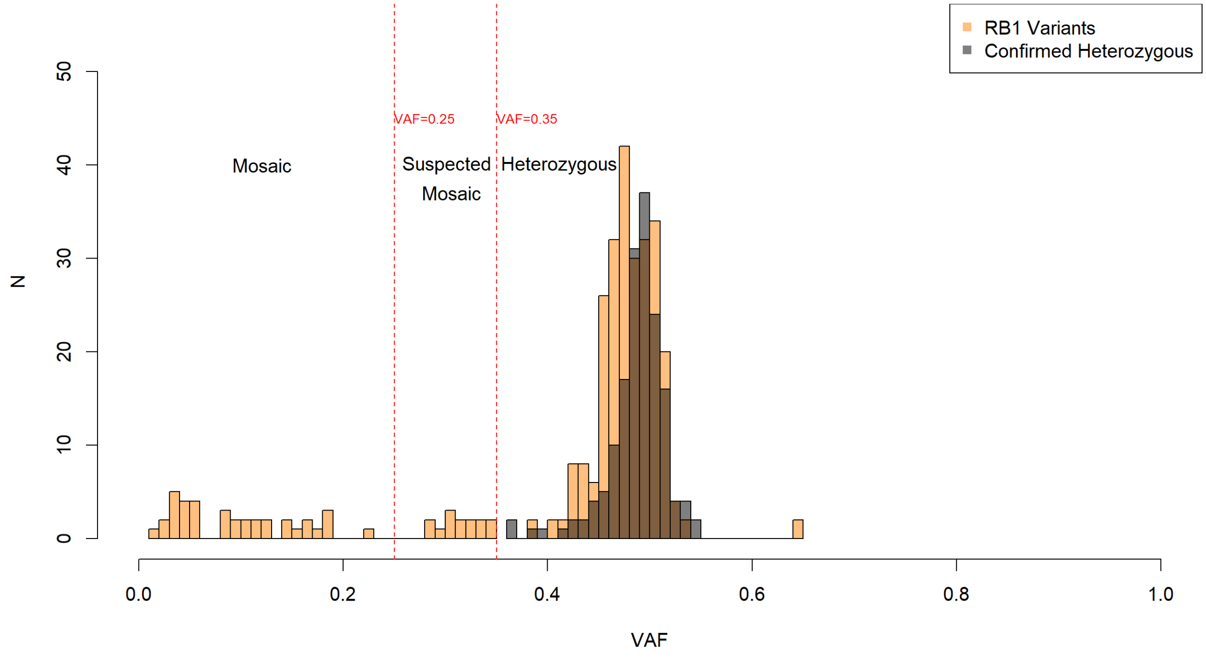

© 2025 Gao C et al. *JAMA Ophthalmology*.

eTable 1. Demographic Table of Study Participants

Variables	Entire Cohort	Mosaic* RB1 variant detected in buffy coat DNA	Heterozygous+ RB1 variant detected in buffy coat DNA	No pathogenic RB1 variant detected in buffy coat DNA
Age at diagnosis, y				
N	136	20 (14.7%)	60 (44.1%)	56 (41.2%)
Mean (SD)	1.37 (1.48)	1.94 (1.85)	0.74 (0.63)	1.82 (1.72)
Min-Max	0.1-9	0.3-8	0.1-2.45	0.1-9
Median (IQR)	1 (0.4-1.69)	1.35 (0.85-2.1)	0.5 (0.25-1.14)	1.35 (0.79-2.15)
Sex				
Female	74 (54.4%)	12 (60%)	30 (50%)	32 (57.1%)
Male	62 (45.6%)	8 (40%)	30 (50%)	24 (42.9%)
Laterality				
Unilateral	69 (50.7%)	9 (45%)	5 (8.3%)	55 (98.2%)
Bilateral	67 (49.3%)	11 (55%)	55 (91.7%)	1 (1.8%)
Ancestry (N)				
Admixed	46	7	19	20
African	7	0	3	4
Asian	12	1	5	6
European	64	12	31	21
Native American	6	0	2	4
Ashkenazi Jewish	1	0	0	1

* Defined as a pathogenic RB1 variant with VAF <35%, including four suspected mosaic INDELs (VAF<35%, and >25%)
+ Defined as a pathogenic RB1 variant with VAF≥35%.

© 2025 Gao C et al. *JAMA Ophthalmology*.

eTable 2. List of All Specimens With Mosaic *RB1* Variants

Participant ID	Specimen Number	c. nomenclature	p. nomenclature	Buffy Coat DNA				cfDNA			
				Total Reads	Ref Reads	Alt Reads	VAF	Total Reads	Ref Reads	Alt Reads	VAF
P1	1	c.1060_1061del	p.Q354Efs*7	552	530	22	0.0399	1155	1119	36	0.0312
P1	2	c.1060_1061del	p.Q354Efs*7	592	560	32	0.0541	269	262	7	0.026
P1	3	c.1060_1061del	p.Q354Efs*7	583	561	22	0.0377	1510	1479	31	0.0205
P2	1	c.1072C>T	p.R358*	757	649	108	0.1427	798	686	112	0.1404
P2	2	c.1072C>T	p.R358*	542	451	91	0.1679	517	467	50	0.0967
P3	1	c.191T>G	p.L64*	768	677	91	0.1185	1878	1643	235	0.1251
P4	1	c.1735C>T	p.R579*	2198	1855	343	0.1561	2591	2269	322	0.1243
P5	1	c.2359C>T	p.R787*	1104	764	340	0.308	1458	1079	379	0.2599
P5	2	c.2359C>T	p.R787*	1981	1411	570	0.2877	1726	1292	434	0.2514
P6	1	c.2237_2241del	p.E746Gfs*3	753	492	261	0.3466	869	580	289	0.3326
P7	1	c.2125_2135dup	p.K713Mfs*6	751	513	238	0.3169	2797	1894	903	0.3228
P7	2	c.2125_2135dup	p.K713Mfs*6	1574	1092	482	0.3062	2525	1723	802	0.3176
P7	3	c.2125_2135dup	p.K713Mfs*6	1513	1056	457	0.302	2596	1809	787	0.3032
P8	1	c.964G>T	p.E322*	1142	974	168	0.1471	3471	2939	532	0.1533
P9	1	c.958C>T	p.R320*	2007	1668	339	0.1689	3827	3152	675	0.1764
P9	2	c.958C>T	p.R320*	1217	1059	158	0.1298	2824	2510	314	0.1112
P10	1	c.2429_2432dup	p.S811Rfs*5	1365	976	389	0.285	3472	2638	834	0.2402
P11	1	c.2363_2384dup	p.R798Qfs*4	1469	1351	118	0.0803	1184	1087	97	0.0819
P12	1	c.1183C>T	p.Q395*	703	680	23	0.0327	1725	1684	41	0.0238
P12	2	c.1183C>T	p.Q395*	937	899	38	0.0406	1075	1028	47	0.0437
P12	3	c.1183C>T	p.Q395*	583	566	17	0.0292	2386	2322	64	0.0268
P12	4	c.1183C>T	p.Q395*	637	620	17	0.0267	2691	2596	95	0.0353
P12	5	c.1183C>T	p.Q395*	640	615	25	0.0391	2150	2083	67	0.0312
P12	6	c.1183C>T	p.Q395*	675	654	21	0.0311	1478	1428	50	0.0338
P13	1	c.1330C>T	p.Q444*	895	854	41	0.0458	734	703	31	0.0422
P13	2	c.1330C>T	p.Q444*	731	700	31	0.0424	2024	1940	84	0.0415
P14	1	c.1174delG	p.A392Qfs*9	976	808	168	0.1721	2628	2127	501	0.1906
P14	2	c.1174delG	p.A392Qfs*9	599	491	108	0.1803	2531	2087	444	0.1754
P14	3	c.1174delG	p.A392Qfs*9	1309	1069	240	0.1833	2672	2182	490	0.1834
P14	4	c.1174delG	p.A392Qfs*9	782	639	143	0.1829	2048	1646	402	0.1963
P15	1	c.45_76del	p.A17Pfs*3	1380	933	447	0.3239	872	508	364	0.4174
P15	2	c.45_76del	p.A17Pfs*3	1023	724	299	0.2923	423	268	155	0.3664
P15	3	c.45_76del	p.A17Pfs*3	941	642	299	0.3177	234	138	96	0.4103
P16	1	c.2073_2075dup	p.Y692*	804	538	266	0.3308	2947	2018	929	0.3152
P16	2	c.2073_2075dup	p.Y692*	891	582	309	0.3468	2208	1579	629	0.2849

© 2025 Gao C et al. *JAMA Ophthalmology*.

P16	3	c.2073_2075dup	p.Y692*	1132	749	383	0.3383	2870	1993	877	0.3056
P16	4	c.2073_2075dup	p.Y692*	900	611	289	0.3211	3872	2802	1070	0.2763
P17	1	c.1399C>T	p.R467*	1121	1029	92	0.0821	2331	1719	612	0.2625
P17	2	c.1399C>T	p.R467*	1375	1255	120	0.0873	2464	2199	265	0.1075
P17	3	c.1399C>T	p.R467*	1063	957	106	0.0997	2107	1938	169	0.0802
P17	4	c.1399C>T	p.R467*	1303	1169	134	0.1028	1455	1337	118	0.0811
P17	5	c.1399C>T	p.R467*	1013	921	92	0.0908	2050	1886	164	0.08
P17	6	c.1399C>T	p.R467*	1781	1586	195	0.1095	2373	2162	211	0.0889
P17	7	c.1399C>T	p.R467*	1413	1243	170	0.1203	3271	2962	309	0.0945
P17	8	c.1399C>T	p.R467*	1473	1302	171	0.1161	2609	2357	252	0.0966
P18	1	c.1735C>T	p.R579*	1946	1858	88	0.0452	2349	2249	100	0.0426
P18	2	c.1735C>T	p.R579*	1774	1682	92	0.0519	2153	2061	92	0.0427
P18	3	c.1735C>T	p.R579*	1447	1374	73	0.0504	3022	2890	132	0.0437
P19	1	c.2359C>T	p.R787*	1410	1393	17	0.0121	3339	3240	99	0.0296
P20	1	c.54_76dup	p.P26Rfs*47	1905	1587	313	0.1643	725	558	166	0.22897

© 2025 Gao C et al. *JAMA Ophthalmology*.

eTable 3. Summary of MSK-IMPACT and External Testing Results for the Identified Mosaic Patients

Patient ID	c. Nomenclature	p. Nomenclature	MSK-ACCESS VAF (%)	MSK-IMPACT VAF (%)	External Testing Results (year)
P1	c.1060_1061del	p.Q354Efs*7	4.39	NA	Reported as mosaic (2009)
P2	c.1072C>T	p.R358*	15.53	18.3	Reported as mosaic (2017)
P3	c.191T>G	p.L64*	11.85	10.6	Negative (2009)
P4	c.1735C>T	p.R579*	15.61	NA	Reported as mosaic (2012)
P5	c.2359C>T	p.R787*	29.79	NA	Reported as heterozygous (2010)
P6	c.2237_2241del	p.E746Gfs*3	34.66	38.4	Reported as heterozygous (2011)
P7	c.2125_2135dup	p.K713Mfs*6	30.84	32.4	Variant detected, zygosity not mentioned (2016)
P8	c.964G>T	p.E322*	14.71	NA	NA
P9	c.958C>T	p.R320*	14.94	NA	Reported as heterozygous (2018)
P10	c.2429_2432dup	p.S811Rfs*5	28.5	27.6	Reported as heterozygous (2018)
P11	c.2363_2384dup	p.R798Qfs*4	8.03	30.8	NA
P12	c.1183C>T	p.Q395*	3.58	NA	Negative (2020)
P13	c.1330C>T	p.Q444*	4.41	3.7	Negative (2019)
P14	c.1174del	p.A392Qfs*9	17.97	16.9	Negative (2020)
P15	c.45_76del	p.A17Pfs*3	31.13	NA	Variant detected, zygosity not mentioned (unclear)+
P16	c.2073_2075dup	p.Y692*	33.43	NA	Variant detected, zygosity not mentioned (unclear) +
P17	c.1399C>T	p.R467*	10.11	12.6	Reported as mosaic (unclear) +
P18	c.1735C>T	p.R579*	4.92	6%	NA
P19	c.2359C>T	p.R787*	1.21	2.1	NA
P20	c.54_76dup	p.P26Rfs*47	12.08	NA	NA

+ Report detail was not available; results were only briefly mentioned in medical records.

© 2025 Gao C et al. *JAMA Ophthalmology*.
