# Supplementary material for: Detection and Characterization of RB1 Mosaicism in Patients With Retinoblastoma Receiving cfDNA Test
Source: JAMA Ophthalmol. 2025 May 8;143(7):562–8. doi: 10.1001/jamaophthalmol.2025.1079 (PMC12062978; doi:10.1001/jamaophthalmol.2025.1079)
Supplement: Supplement 2. — Data Sharing Statement [file jamaophthalmol-e251079-s002.pdf]

Data Sharing Statement

Gao. Detection and Characterization of RB1 Mosaicism in Patients With Retinoblastoma Receiving cfDNA Test. *JAMA Ophthalmol*. Published May 08, 2025.
doi:10.1001/jamaophthalmol.2025.1079

Data
Data available: Yes
Data types: Deidentified participant data, Other (please specify)
Additional Information: individual level mosaic RB1 variant information from cfDNA and buffy coat DNA
How to access data: individual level data of the mosaic patients have been included in supplemental tables
When available: With publication

Supporting Documents
Document types: None

Additional Information
Who can access the data: anyone who has access to the supplementary tables
Types of analyses: for research purposes
Mechanisms of data availability: with investigator support
